# Lytic and Molecular Evidence of the Widespread Coriander Leaf Spot Disease Caused by *Alternaria dauci*

**DOI:** 10.3390/plants12223872

**Published:** 2023-11-16

**Authors:** Khalid M. Ghoneem, Abdulaziz A. Al-Askar, Seham M. A. El-Gamal, Ehsan M. Rashad, Elsherbiny A. Elsherbiny, Shafik D. Ibrahim, Samy A. Marey, WesamEldin I. A. Saber

**Affiliations:** 1 Department of Seed Pathology Research, Plant Pathology Research Institute, Agricultural Research Center (ARC), Giza 12619, Egypt; khalidghoneem@arc.sci.eg (K.M.G.); ehsanrashad78@arc.sci.eg (E.M.R.); 2 Department of Botany and Microbiology, Faculty of Science, King Saud University, Riyadh 11451, Saudi Arabia; aalaskara@ksu.edu.sa; 3 Department of Medicinal and Aromatic Plants Research, Horticulture Research Institute, Agricultural Research Center (ARC), Giza 12619, Egypt; s_elgamal99@yahoo.com; 4 Department of Biology, Rheinland-Pfälzische Technische Universität Kaiserslautern (RPTU), 67663 Kaiserslautern, Germany; 5 Department of Genome Mapping, Agricultural Genetic Engineering Research Institute (AGERI), Agricultural Research Center (ARC), Giza 12619, Egypt; shafikdarwish@ageri.sci.eg; 6 King Saud University, Riyadh 11451, Saudi Arabia; samarey@ksu.edu.sa; 7 Microbial Activity Unit, Department of Microbiology, Soils, Water and Environment Research Institute, Agricultural Research Center (ARC), Giza 12619, Egypt

**Keywords:** fungal pathogens, lytic enzymes, morphological identification, molecular identification, *Alternaris* gene

## Abstract

*Coriandrum sativum* L. is a globally significant economic herb with medicinal and aromatic properties. While coriander leaf blight disease was previously confined to India and the USA, this study presents new evidence of its outbreak in Africa and the Middle East caused by *Alternaria dauci*. Infected leaves display irregular chlorotic to dark brown necrotic lesions along their edges, resulting in leaf discoloration, collapse, and eventual death. The disease also impacts inflorescences and seeds, significantly reducing seed quality. Koch’s postulates confirmed the pathogenicity of the fungus through the re-isolation of *A. dauci* from artificially infected leaves, and its morphology aligns with typical *A. dauci* features. Notably, this study identified strong lytic activity (cellulase: 23.76 U, xylanase: 12.83 U, pectinase: 51.84 U, amylase: 9.12 U, and proteinase: 5.73 U), suggesting a correlation with pathogenicity. Molecular characterization using ITS (ON171224) and the specific *Alt-a-1* gene (OR236142) supports the fungal morphology. This research provides the first comprehensive documentation of the pathological, lytic, and molecular evidence of *A. dauci* leaf blight disease on coriander. Future investigations should prioritize the development of resistant coriander varieties and sustainable disease management strategies, including the use of advanced molecular techniques for swift and accurate disease diagnosis to protect coriander from the devastating impact of *A. dauci*.

## 1. Introduction

Coriander, known as cilantro or Chinese parsley, is scientifically labeled as *Coriandrum sativum* L. and belongs to the *Apiaceae* family. This herb is an annual plant native to Egypt, Turkey, and the eastern Mediterranean Sea. Coriander is cultivated as a profitable cash crop, predominantly in arid and semi-arid areas of Egypt during the winter season. The major global producers of coriander include India, Morocco, Pakistan, Bulgaria, Romania, Canada, the former Soviet Union, Ukraine, and Syria. Additional producers encompass Iran, Turkey, Israel, Egypt, China, the USA, Argentina, and Mexico. Notably, India contributes to over 80 percent of the total coriander production [1,2,3].

In Egypt, between the years 2000 and 2017, coriander exports amounted to 61.13 thousand US dollars, generated from a cultivated area of around 9500 acres. This represents 12.6% of the total cultivated area dedicated to medicinal and aromatic plants. According to competitiveness indicators, Egyptian coriander exportation is expected to rise from 407.6 tons in 2017 to 893.6 tons in 2022, reflecting a substantial 119.2% increase [4].

Both its leaves and seeds of coriander have a variety of uses, such as cooking, perfumes, cosmetics, and medicine [5]. Additionally, the essential oils derived from its seeds have been found to have properties such as carminative, laxative, anti-diabetic, diuretic, hypolipidemic, and anti-cancer actions. Nutritionally, coriander is a great supply of calcium, iron, vitamin C, and provitamin A [6,7].

Several fungal pathogens are responsible for causing leaf spot diseases on coriander plants worldwide, such as *Ulocladium oblongoobovoideum*, *Alternaria alternata*, *Colletotrichum gloeosporioides*, *Colletotrichum capsica*, *Cercospora corianderi*, *Phoma multirostrata*, and *Cladosporium tenuissimum* [8,9,10,11]. Among phytopathogens, the genus *Alternaria* is one of the most important, comprising a great number of pathogens that lead to large-scale losses in several crops [12,13]. 

Most *Alternaria* spp. survive as a mycelium or spores in dead host tissue left on or in the soil, and on or within seeds from infected plants, which are spread from it by wind or splashing water. In this respect, seeds represent the main means of transmission of some *Alternaria* diseases, causing the rotting of seeds or infection of radicals, and finally seedling mortality [7,12,14,15,16]. Under favorable conditions, *Alternaria* disease symptoms are characterized by the emergence of brown lesions on the different infected plant tissues. On leaves, for example, the disease progresses and when approximately 40% of the leaf surface is affected, the entire leaf turns yellow and dies, resulting in the characteristic blights [7].

*Alternaria alternata*, *A. dauci*, and *A. radicina* have been reported as large-scale phytopathogens in the *Apiaceae* family, which can attack different plant parts causing lesions on leaves, petioles, and inflorescences, as well as attack the roots leading to seedling damping-off [7]. Several *Alternaria* spp. have been recorded on *C. sativum* [17]. *Alternaria poonensis* (syn. *A. dauci* (Kuhn) Groves and Skolko) was first defined in Puerto Rico [18] and later reported in some other parts of the world, e.g., Mauritius [19]. In the following 14 years, *A. dauci* has been reported in many other countries including India [20,21], the United States [22], Algeria [23], and recently in Brazil [24]. Coriander plants and seeds have recently been found to be infected with *A. dauci* [7,25].

*Alternaria dauci* was reported as a polyphagous pathogen infecting several plants belonging to *Apiaceae*, including carrots, parsley, and celery [24]. It has also been claimed that *A. dauci* can infect wild parsnip, celery, and parsley [12].

Identifying *Alternaria* species can be challenging due to intraspecific polymorphisms and interspecific similarities, compounded by their sensitivity to environmental variations, which can introduce uncertainty when solely relying on phenotype-based taxonomy. Traditionally, identification involved morphological features, such as conidial shape, primary conidium size, conidial branching arrangement, and primary conidiophores length as described by Simmons and Roberts [26]. However, this morphological and cultural approach often proves to be intricate, as highlighted by Simmons [27]. Therefore, to effectively identify *Alternaria* species, a combination of both morphological and molecular protocols is essential.

The molecular technique could therefore be useful for the characterization of *Alternaria* spp. [28,29]. A vital step in the identification of *Alternata* spp. is the use of DNA analysis techniques such as the PCR-based technique, DNA sequencing, and gel electrophoresis. Because of the overlapping characteristics, it can often be difficult to differentiate between species, especially those in *Alternaria.* The *Alt-a-1* gene sequence helps to identify *Alternata* species by identifying characteristics and phylogenetic analyses of the main allergen [28,29,30].

From November 2020 to March 2021, many coriander plants grown in the research farms of Tagelez and El-Baramoon, located in Dakahlia governorate, Egypt, were found to have severe foliar blight. This was characterized by the appearance of dark brown, oval, or irregularly shaped lesions on the edges of leaves, which eventually turned fully yellow, collapsed, and died. Yellow halo lesions on the leaves and petioles were also observed. Brown spots were also seen on the stems and branches, and the umbels showed neck blight, which ultimately led to the death of the plant. This disease caused a decline in the overall health and/or seed quality of the plants.

The symptoms observed in coriander plants, suggestive of *Alternaria* disease, mark a significant revelation in the context of Egyptian agriculture. To the best of our knowledge, before this study, *Alternaria* disease had not been reported. This represents a fundamental shift in the understanding of plant diseases in the region and underscores the importance of our research. The novelty of this work extends to the molecular and lytic description of *A. dauci* on coriander. While other studies may have documented *Alternaria* species in different contexts, the detailed molecular characterization and lytic description of this pathogen on coriander plants are unprecedented. Perhaps most notably, this study presents a pioneering effort in isolating and confirming *A. dauci* as the causative pathogen using a molecular approach. The use of the internal transcribed spacer (ITS) region and *Alt-a-1* gene not only provides a robust means of identification but also serves as a milestone in plant pathology research. In addition, the extensive exploration of the enzymatic profile as a potential infection route adds another layer of novelty to our work. Understanding the enzymatic mechanisms involved in the pathogenicity of *A. dauci* is a crucial step in developing effective disease management strategies.

The primary objective of this study is to identify and confirm *A. dauci* as the causative pathogen responsible for the observed symptoms in coriander plants in Egypt. To provide a comprehensive understanding of the pathogenicity of *A. dauci*, the study aims to conduct extensive morphological, molecular, and lytic descriptions. A further aim is the exploration of the enzymatic profile of *A. dauci* as a potential route of infection. This will involve identifying and characterizing enzymes associated with the pathogen’s pathogenicity. Another key objective is through molecular techniques, specifically targeting the ITS region and the *Alt-a-1* gene.

## 2. Materials and Methods

### 2.1. Disease Observation and Isolation of the Pathogen

From November 2020 to March 2021, we collected infected coriander samples exhibiting severe foliar blight from two distinct locations: the research farm of the Tagelez research station (30°57′25″ N and 31°35′54″ E) and El-Baramoon Farm, Mansoura Horticulture Research Station (31°08′11.3″ N and 31°28′19.6″ E) in Dakahlia governorate, Egypt. A total of 200 coriander plant samples were obtained from each location, and each set of samples was separately processed to assess the pathogenicity and aggressiveness of the pathogen, enabling the identification of the most virulent phytopathogenic fungal isolate.

In order to isolate pathogens, sections of diseased plant tissue measuring 5 to 10 mm^2^ were first treated with sodium hypochlorite (2%) for 2 min, then washed thrice with sterilized distilled water, dried, and placed on potato sucrose agar (PSA) medium (Difco, Detroit, MI, USA), pH 6.5, which also included streptomycin sulfate (0.3 mg/L) and L-chloramphenicol (0.1 mg/L) as antibacterial agents. After incubation (7 days, 20 ± 2 °C) under fluorescent, cool-white light with a 12-h cycle of photoperiod, the morphology of the recovered fungi was identified using a light-supported stereomicroscope (Olympus SZ2-ILST, Shinjuku, Tokyo, Japan) and light microscopy (Olympus CX41, Shinjuku, Tokyo, Japan). 

### 2.2. Purification of the Pathogen

The hyphal tip procedure was applied to purify fungal pathogens. The tip of fungal hyphae (thin, thread-like structures in fungi) was selected as an active single and pure fungal strain. The selected hyphal tip was placed on potato dextrose agar (PDA) medium (Difco, Detroit, MI, USA). The pure colony recovered from the grown hyphal tip was transferred to potato carrot agar (PCA) medium and identified. The slants with pure fungal colonies were then stored at 4 °C to maintain cultures for future studies.

### 2.3. Pathogenicity and Assessment of A. dauci Aggressiveness

Fungal pathogenicity testing was conducted using pots with a 40-cm diameter, filled with 8 kg of steam-sterilized soil (comprising 33% clay, 25% sand, and 41.5% silt). Healthy-looking coriander seeds (Balady) were surface sterilized by applying 1% NaClO for 2 min, followed by rinsing with sterilized water and air drying. Six coriander seeds were then planted in each pot, and after germination, the seedlings in each pot were thinned to two plants. All pots were maintained under greenhouse conditions for three months, with temperatures at 25 ± 2 °C, a 16-h photoperiod, and relative humidity ranging from 80% to 85%. 

To evaluate the pathogenicity, the fungal inoculum was prepared by growing the 2 fungal isolates (Ad2022 and Ad2023) in V8 juice agar medium (200 mL V8 vegetable juice, 3 g CaCO_3_, 15 g agar L^−1^, pH 6.8). The plates were incubated (15 days, 25 ± 2 °C) under fluorescent, cool-white light with a 12-h light/12-h darkness alternating cycle.

For each isolate, the growing culture was carefully covered with sterilized water, to harvest the conidia with a glass rod. The resulting fungal suspension was mixed with 0.05% tween 20. Using a hemocytometer, a concentration of 10^5^ conidia/mL was prepared and sprayed onto 3-month-old plants. The control group was sprayed with sterilized water. Inoculated and nonincubated plants were enclosed for 2 days in plastic pots to keep high relative moisture. A total of 100 plants (pots experiment, two plants/pot) were inoculated. The plants were then observed for 20 days [24].

The disease incidence (the infected plants divided by the total number of examined plants, with a sample size (*n*) of 100) was calculated. The severity of the disease was visually rated using the scale of Pawelec et al. [31]. The severity of the disease was estimated by the percentage of necrotic leaf area (0: no visible symptoms, 1: <5% leaf area affected, 2: 5% ≤ leaf area affected <20%, 3: 20% ≤ leaf area affected <40%, 4: 40% ≤ leaf area affected <60%, and 5: ≥60% leaf area affected). The mean area of lesions at 15 and 20 days post-inoculation (dpi), the mean disease index (an indicator of aggressiveness), and the necrotic leaf area % at 20 dpi were calculated. The experiment was repeated twice for accuracy.

### 2.4. Enzymatic Profile of A. dauci

To investigate the fungus’s ability to break down and utilize the coriander plant material as a source of nutrients and energy, the pathogenic fungus was assessed for various catalytic actions on a solid-substrate fermentation (SSF) medium. The dried coriander plant was ground and used as a substrate in the fermentation medium.

#### 2.4.1. Culturing Conditions

One gram of dried plant was mixed with 5 mL of tap water (pH 6.5) in a 250 mL Erlenmeyer flask and sterilized at 121 °C for 15 min. The flask was injected with 1.0 mL of conidia suspension. The SSF moisture content of the medium was kept constant at 65%, and incubated for 10 days at 30 °C, followed by the addition of 10 mL of Tween 80 (0.01%) and agitated for 30 min (150 rpm). Finally, the fungal filtrate was separated at 5000 rpm for 20 min and analyzed for the lytic enzymes (cellulase, xylanase, pectinase, amylase, and proteinase).

#### 2.4.2. Assay of Lytic Enzymes

The post-culture filtrate was tested for cellulase and xylanase activity by using microcrystalline cellulose [32] and xylan [33] as substrates, respectively. The substrates were thawed at a concentration of 0.5% in a citrate buffer at 0.05 M, pH 4.8, and then 1 mL of the fungal filtrate and 1 mL of the substrate-buffer mixture were mixed and incubated at 50 °C for 60 min for the cellulase assay and 30 min for the xylanase assay. The activity of pectinase was measured by incubating the fungal filtrate with the sodium acetate buffer (pH 5.2) for 30 min at 40 °C [34].

The α-amylase activity was measured by combining the enzyme with 0.5% starch in a phosphate buffer at pH 6.5 and then incubating the mixture at 30 °C for 10 min [35].

The previous enzymatic actions were calculated in the enzyme unit by determining the amount of liberated reducing group [36]. One unit of each enzyme (U) was described as the enzyme amount needed to liberate 1.0 µmol/g/min of glucose (for cellulase and amylase), xylose (for xylanase), and polygalacturonic acid (for pectinase) under the reaction conditions. This activity was determined using standard curves.

The activity of protease was tested by measuring the released amino acids from casein using fungal filtrate at 37 °C for 10 min. The released amino acids were quantified at *A*_280_ [37]. The unit of the enzyme was the amount that resulted in one µg of tyrosine equivalent/g/min under the reaction situations.

### 2.5. Morphological Identification

The pathogenic, *A. dauci* conidia (*n* = 125), was identified by scraping the colony in the presence of 20 mL of distilled water, and then a microscopic slide was prepared. The conidial morphology was investigated under the light microscope (Leica DM4500B; Leica Microsystems, Wetzlar, Germany). The cultural features, fungal morphology, and microscopic characteristics were identified according to Simmons [27].

### 2.6. Molecular Identification and Phylogenetic Analysis

#### 2.6.1. ITS Ribosomal RNA Analysis

Molecular identification was performed based on ITS ribosomal RNA analysis. The ITS1 (5′ TCTGTAGGTGAACCTGCGG 3′) and the ITS4 (5′ TCCTCCGCTTATTGATATGC 3′) primers were used. The PCR mixture used in this experiment contained 1x buffer (Promega, Madison, WI, USA), 15 mM MgCl_2_, 0.2 mM dNTPs, 20 picomoles of each primer, 1 µ of Taq DNA polymerase (GoTaq, Promega), 40 ng DNA, and ultrapure water to a total volume of 50 µL. The PCR amplification was carried out using a PerkinElmer/GeneAmp PCR System 9700 (PE Applied Biosystems, Waltham, MA, USA), with 35 cycles of denaturation (95 °C), annealing at a specific temperature, and elongation at 72 °C, followed by a final extension segment of 7 min (72 °C). The resulting PCR product was then aligned with the ITS rRNA sequences of the representative type strains of the genus *Alternaria* using the NCBI-BLAST tools (http://www.ncbi.nlm.nih.gov/BLAST, accessed on 1 March 2022). Three samples of the DNA were sequenced, and the result of the three sequences showed no differences among them, thus only one sequence was submitted. The evolutionary study of the PCR product was deduced according to the Neighbor-Joining method [38]. The evolutionary distances were calculated using the Jukes and Cantor [39] method, using a bootstrap of 1000 replications, which is based on the number of base substitutions per site. This analysis involved 26 nucleotide sequences. Any positions that were not clear were omitted based on pairwise deletion. MEGA 11.0.13 was used for evolutionary analysis.

#### 2.6.2. *Alt-a-1* Gene Analysis

The specific gene sequencing analysis (*Alt-a*) of *A. dauci* was analyzed. Amplification of the PCR was carried out in a mixture of 25 μL Master Mix, 2μL primer F (Alt-F, 5′ ATGCAGTTCACCACCATCGC 3′), 2 μL primer R (Alt-R, 5′ ACGAGGGTGAYGTAGGCGTC 3′) (10 picomoles of each primer), 3μL template DNA (10 ng), and 15 μL dH_2_O [28]. The gene sequence was aligned with other gene sequences in GenBank using the BLAS tool. Aligning the sequences from each locus ensured that corresponding positions in the sequences were correctly matched. The MAFFT tool was used for multiple sequence alignments (30 sequences). The evolutionary inference method (using a bootstrap of 1000 replications) was applied to sequential or individual spot data. The common method of the neighbor-joining method was used. The phylogenetic tree was visualized using MEGA11.

## 3. Results and Discussion

### 3.1. Isolation and Pathogenicity Test

#### 3.1.1. Isolation of the Fungal Pathogen

Between November 2020 and March 2021, a significant foliar blight outbreak occurred in coriander plants at the research stations of Tagelez and El-Baramoon in the Dakahlia governorate. The disease exhibited a variable incidence ranging from 30% to 50% and a severity level ranging from 15% to 43%, with *n* = 100. Two causal pathogens responsible for coriander leaf spots were isolated from the infected plants and subsequently purified. To determine the most aggressive phytopathogenic isolate for further investigations, a pathogenicity test was conducted on healthy plants following Koch’s postulates. This test aimed to identify the isolate with the highest pathogenicity.

#### 3.1.2. Pathogenicity Test

**Figure 1:**
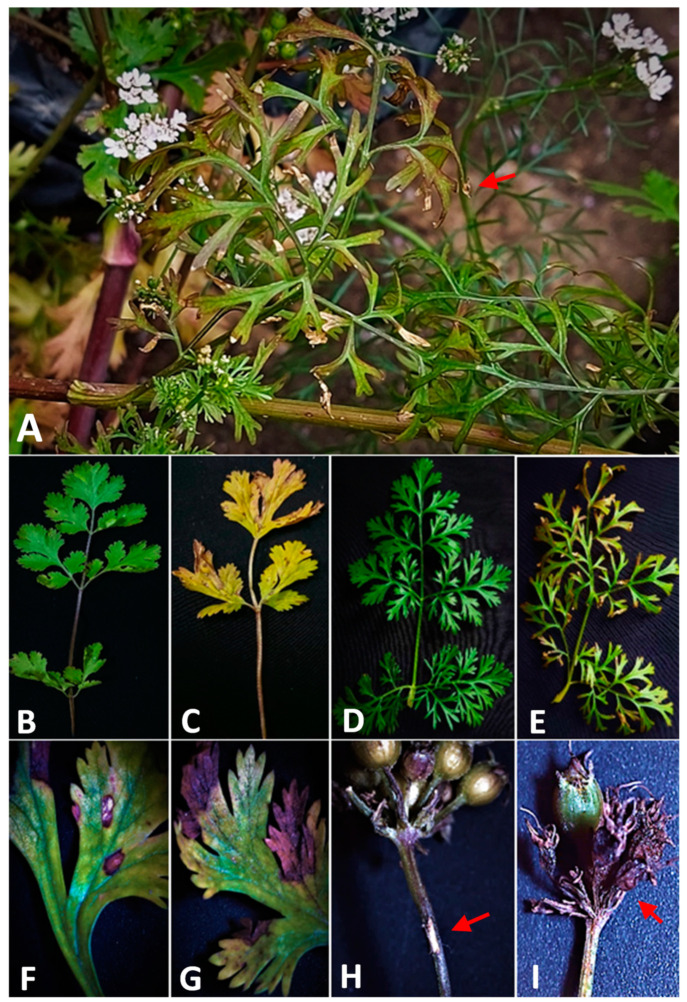
Disease symptoms on coriander plants caused by artificial inoculation with *Alternaria dauci*. (**A**) leaf blight and stem necrosis (arrow), (**B**) healthy young leaf, (**C**) oval or irregular dark brown lesions on margins and tips of coriander leaflets surrounded by a chlorotic halo, (**D**) healthy mature leaf, (**E**) small brown water-soaked spots appear on leaflet surrounded by a well-defined yellow halo, (**F**,**G**) close-up on infected leaf 21 days after infection, and (**H**,**I**) close-up on short-stalk umbels, showing a brown and irregular lesion and blighted umbel (arrowed).

The pathogenic isolate was used to artificially infect 3-month-old plants, and after 7 days up to three weeks, the infected plants displayed typical symptoms such as those observed in the field (Figure 1A). These symptoms included elongated brown lesions on the plant leaf, with different severity rates. The severity of the infection was measured by the leaf necrotic area, which ranged from 21% to 86% (Figure 1B–G). The control plants, on the other hand, remained symptomless.

Symptoms on the infected plants first appeared on older leaves as irregular or oval dark brown lesions at the boundaries of leaves, which are fully yellow, collapse, and die during extended moist and warm weather. Lesions also appeared on coriander short-stalk umbels and had a distinctive chlorotic, yellow halo. The blight also appeared on the stems and branches (Figure 1H,I) as brown blotches. The umbels showed neck blight, which led to their death. Koch’s postulates were fulfilled as the fungal pathogen was successfully re-isolated from 100% of the symptomatic leaves, out of a total of 50 leaves tested. This confirms the causal relationship between the pathogen and the symptoms observed on the infected plants.

*Alternaria dauci* has been reported as a causal pathogen of cultivated and wild carrots and it has also been claimed that it can infect wild parsnip, celery, and parsley [12,24]. The suitability of other alternate hosts, such as *Ridolfia segetum* (corn parsley) and *Caucalis tenet* (hedge parsley), to be infected by *A. dauci* below controlled environment conditions was reported [40].

Certain *Alternaria* spp. have been documented to produce zinniol, a phytotoxin, and enzymes that degrade plant cell membranes and chloroplasts, ultimately resulting in the chlorotic symptoms characteristic of the disease [12,13]. Notably, in the case of *A. dauci* cultures, at least seven phytotoxins have been isolated and characterized, including zinniol, alternariol, alternariol monomethyl-ether, α-acetylorcinol, p-hydroxybenzoic acid, and aldaulactone [41,42,43,44,45,46]. These compounds may potentially play a role in the pathogenicity of *A. dauci* [46].

### 3.2. Morphological Identification

**Figure 2:**
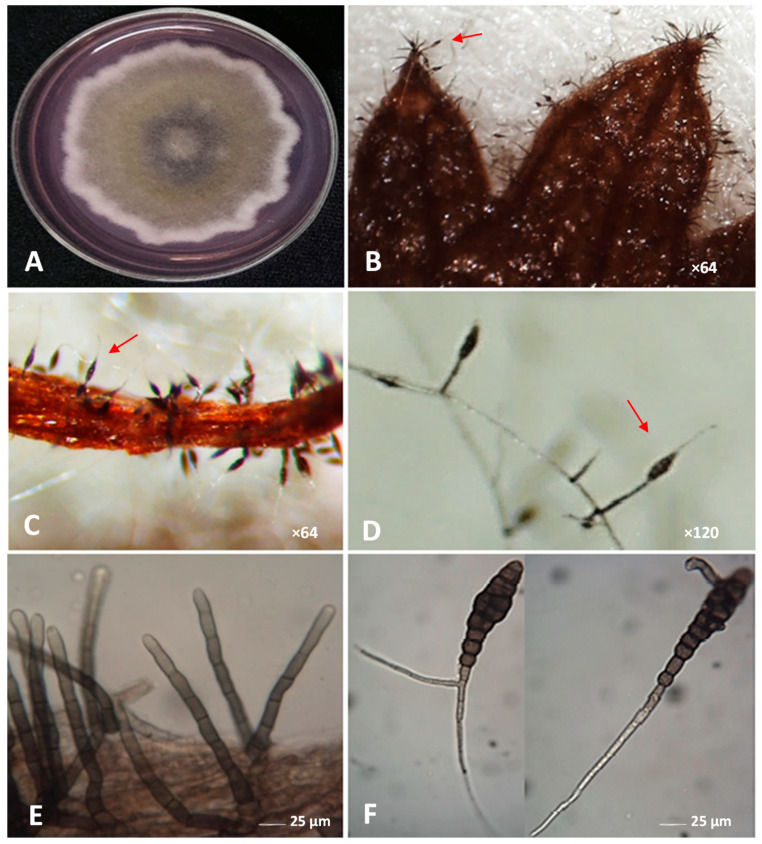
The fungal colony of *Alternaria dauci* on PSA medium with diffusible pink pigment (**A**), stereoscopic micrograph of the fungus on coriander leaf surface completely covered by conidiophores bearing conidia (**B**,**C**), and their close-up (arrows) (**D**), the microscopic photograph showing conidiophores arising on the hyphae ((**E**), ×400), and typical conidia of the fungus ((**F**), ×400).

The morphological features of two isolates (Ad2022 and Ad2023) were explored. Both pathogens produced gray colonies (53.5 to 62.1 mm in diameter), with cottony mycelia and white peripheries on V8 juice medium at 25 ± 2 °C. On PSA, the colony had a diffusible pink pigment after 7 days of incubation (Figure 2A). 

The conidiophores (Figure 2B–E) were straight, measuring 81.6 × 6.2 µm (68.0 to 104 × 6.0 to 6.2 µm, *n* = 120) with six to nine transverse septa, pale brown to brown, and smooth. The conidiophores bore solitary conidia, which were light to dark brown, with a body size of 92.76 × 28.38 µm (62 to 130.2 × 24.8 to 34.1 µm, *n* = 150), and a long-tapered beak (99.2 to 279 µm), pale brown, walls somewhat verruculose, i.e., having small warts or pimples.

Conidia with two beaks were also noticed (Figure 2F). The transverse septa and longitudinal septa of conidia measured 6 to 9 and 0 to 4 μm, respectively. 

This statement suggests that the current findings are consistent with the findings of the previously conducted study but with slightly different measurements. The conidiophores measurements were two to eight transverse septa with a length of 45.2–170 µm (average; 81.8), a width of 5–10 µm (average; 7.2), and the body size of mature conidia of 45.2–92.9 × 12.5–30.1 (average; 68.3 × 18.4) µm with a beak up 301.4 µm long, further the transverse-septa and longitudinal-septa of conidia measuring 10–18 and 0–4 μm, respectively [23]. 

Similar approximate results [24] on coriander reported some differences in conidiophores measurements of *A. dauci*, being 27.5–75 × 5–10 μm in length and 1–4 μm septate, and conidia solitary with a body size of 200–300 × 17.5–22 μm, longitudinally 1–8 and 8– transversally 15 μm septa, with a beak up to 280 μm long. The pathogens were identified as *A. dauci* based on their morphological characteristics [27].

### 3.3. Lytic Activity Profile of A. dauci

**Figure 3:**
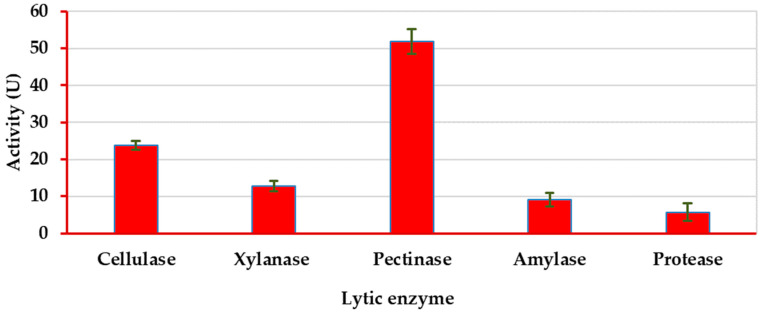
Enzymatic profile of the pathogenic *Alternaria dauci* on coriander plant tissue (U mean ± SD).

We chose the most virulent and aggressive pathogenic fungus, *A. dauci* Ad2022, isolated from the Tagelez location, for a comprehensive enzymatic system analysis. This study aimed to elucidate the mechanisms by which this fungus degrades plant tissues through hydrolysis and to pinpoint the specific enzymes responsible for the invasion process (Figure 3).

The study revealed that *A. dauci* exhibits substantial activity of diverse hydrolytic enzymes, with values of 23.76 U for cellulase, 12.83 U for xylanase, 51.84 U for pectinase, 9.12 U for amylase, and 5.73 U for proteinase. These findings suggest its capacity to effectively break down plant tissue through hydrolysis.

This test had a specific focus on assessing the activity of particular enzymes in *A. dauci* that are recognized for their role in breaking down plant cell walls to clarify the fungal pathogen’s capacity to infiltrate coriander plant tissues. These enzymes play a crucial role in the breakdown of plant tissues at the infection site, constituting the initial stage of the invasion process [37,40]. 

The current data indicated the enzymatic breakdown of plant tissues, which established a direct connection between the lytic activity of *A. dauci* and its pathogenicity. This implies that the fungus’s capability to degrade plant tissues is a critical factor in the development of the disease it induces [37,47]. 

The complex structure of plants makes it difficult for phytopathogens to invade, so enzymes that break down plant tissue, called lytic enzymes (cellulase, xylanase, pectinase, amylase, and protease), are crucial for the pathogenesis process [28,29]. It was suggested that there is a significant difference in the enzymatic profile of virulent and avirulent bacteria, which may be related to their ability to cause disease [32,48]. This is why lytic enzymes are so important in the progression of the disease [32,48,49]. However, the determination of the enzymatic profile could be considered a means of understanding how the fungus causes disease and potentially developing ways to control or prevent it.

Plant cellulose is a complicated carbohydrate that is a major constituent of plant cell walls. Cellulolytic activity breaks down cellulose by hydrolyzing the bonds between the glucose units that make up cellulose [37,47]. This process results in the release of individual glucose monomers, which can be used by the fungus as a source of energy. Cellulase is one of the key enzymes included in the lytic activity of the fungus and in its ability to penetrate plant tissue [32,37,47]. 

The study found that *A. dauci* has a moderate level of xylanase activity, which is an enzyme that breaks down xylan, a component of plant cell walls known as hemicellulose. The presence of xylanase activity suggests that the fungus can degrade xylan, and thus, break down the hemicellulose component of plant tissue, this can facilitate the invasion of the fungus into the plant tissue and the progression of the disease [37,50,51]. 

Pectin is another complex polymer of carbohydrates that is also a constituent of plant cell walls. The pectinolytic enzyme breaks down pectin by splitting the α-1,4-glycosidic bond between the galacturonic acid units that make up pectin. This process results in the release of individual galacturonic acid monomers, which can be used by the fungus as a source of energy [34,47]. Pectinase is one of the enzymes involved in the lytic activity of the fungus and in its ability to penetrate plant tissue. Pectinase also plays a vital task in the pathogenicity process by breaking down the pectin component of the plant cell walls [34,47]. 

Starch is a complex carbohydrate that is stored in plant cells as a reserve of energy. Amylase is an enzyme that breaks down starch by hydrolyzing the bonds between the glucose units that make up starch. This process results in the release of individual glucose units, which can be used by the fungus as a source of energy. Amylase is another key enzyme involved in the lytic activity of the fungus and in its ability to utilize starch as a source of energy [52]. 

Proteases break down the nitrogen-containing compounds in the plant cell wall into peptides and amino acids, these smaller compounds can then be used by the fungus for growth and various metabolic processes or stored for later use [52].

The group of enzymes in this consortium synergistically work to break down plant tissue, making it easier for the pathogen to infect the plant. These lytic enzymes also pose a significant threat to the plant’s health [40]. Overall, the pathogen has a combination of enzymes that can lead to severe infection.

### 3.4. Molecular Identification

The current study primarily aimed to study the genetic variation of *A. dauci* using two genes, i.e., ITS ribosomal RNA and *Alt-a-1* gene analysis.

#### 3.4.1. Identification of *A. dauci* Based on ITS Analysis

**Figure 4:**
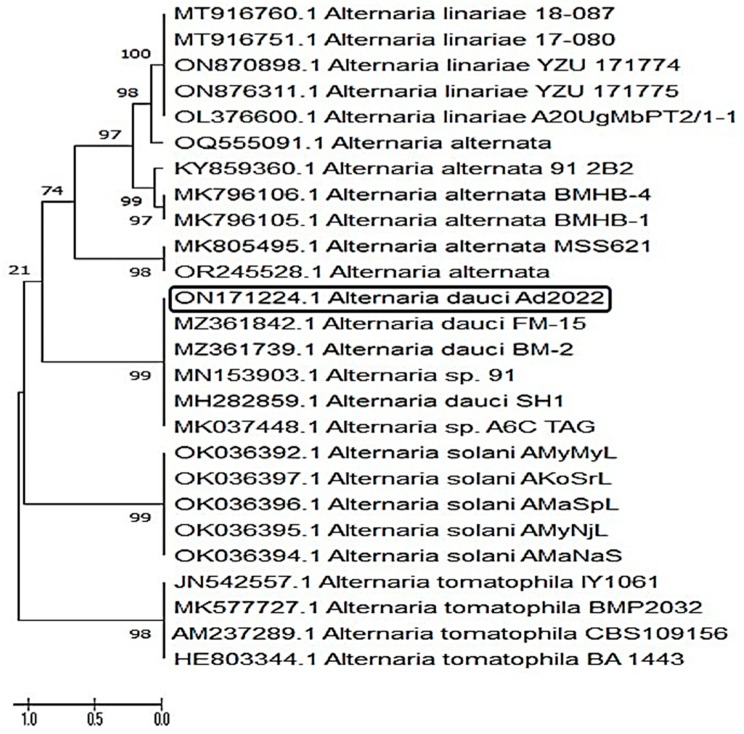
A phylogenetic tree, using sequence analysis of a concatenated sequence from the ITS gene, shows the *Alternaria dauci* (GenBank number; ON171224, in black box) among the similar sequences *Alternaria* spp. found in the GenBank database. Bootstrap of 1000 replications; external group.

*Alternaria dauci* was molecularly identified based on ITS ribosomal RNA. Alongside PCR amplification, the phylogenetic analysis was performed and the sequence, which codes for the 18S rRNA gene, was compared. Based on the BLAST and phylogeny analysis (Figure 4), *Alternaria dauci* exhibited high resemblance, up to 99%, with the formerly representative *A. dauci* on the GenBank, confirming previous morphological data.

The sequence obtained from amplifying the ITS fragment indicated that the target sequence shares similarities with fungal sequences found in the GenBank database. Upon conducting a multi-gene phylogenetic analysis, the pathogenic isolate matched with related sequences from the GenBank. It was observed that the resulting phylogenetic tree is divided into two main clads (clusters). The first clade is divided into two subclusters, containing all *Alternaria* spp., including our isolate. From the phylogeny analysis, it is obvious that our isolate is closely similar to the other *A dauci* (MZ361824.1 and MZ361739.1) of the same subcluster. Accordingly, the GenBank accession number was received as ON171224.1.

Phylogeny analysis is a method used in evolutionary biology to infer the evolutionary relationships among a group of organisms based on genetic information. This approach is particularly valuable when studying complex evolutionary histories or when a single gene may not provide enough resolution to accurately reconstruct the relationships [53,54,55].

Despite the ongoing advancements in molecular techniques and their increasing availability, traditional methods such as microscopic and cultural techniques are still widely used and indispensable for fungal identification. For instance, morphological methods, including macroscopic and microscopic characteristics are, particularly, the most reliable and sensitive means of identifying *Aspergillus* species isolated from environmental and clinical specimens [56,57]. However, the molecular technique of ITS was utilized together with the morphological classification to confirm each other. 

The ITS technique is a sensitive and specific, rapid, and accurate identification tool of fungi. The ITS region is consistent across many different types of fungi, making it useful for identifying both differences between different species of fungi and variations within a single species [58,59]. Furthermore, the sequence of such non-functional areas is extremely miscellaneous among fungal species, which makes it useful for identifying fungal species [59]. Practically, the multi-copies of the rDNA of the ITS regions make it easy to amplify from a minute sample of DNA [60]. Consequently, using the ITS region for nucleotide sequencing is considered an efficient and accurate method for identifying a wide range of fungi at the species level. Therefore, nucleotide sequencing of ITS is considered among the markers with a quick and uppermost probability of precise identification as well as barcode identification [61,62]. 

This work confirms that *A dauci* was the causative pathogen of leaf blight on coriander for the first time in Egypt. Thus, it could be deduced that the appearance of this disease in new regions sounded the alarm to warn of the acceleration of the spread of the disease, especially under the global change in climatic conditions in the recent period. 

Environmental conditions are a crucial factor in plant pathology, representing one side of the “disease triangle” alongside the pathogen and the host [63]. In this connection, extreme changes in climatic conditions, even for a short time, may significantly affect growth, survival, resistance, and plant health [64], which may generally, suppress, or promote the growth, toxicity, virulence, host specificity, reproduction, dissemination, or geographic distribution of the pathogens, thus affecting disease severity and spread [65]. This explanation could be the reason for the spread of the newly detected *A dauci* in the current study.

#### 3.4.2. Identification of *A. dauci* by *Alt-a-1* Gene Analysis

The molecular identification of *A. dauci* using the target sequence for the *Alt-a-1* gene. The sequences *Alt-a-1* have been submitted to the NCBI and have been assigned to gene bank accession numbers (OR236142). The results of the BLAST matching analysis of *Alternaria dauci* with the GenBank isolates with the highest similarity percentages were compared (Table 1). Newly generated sequences of the *Alt-a-1* gene were aligned with the current *Alt-a-1* gene sequences in GenBank accessions, which displayed a query coverage between 94 to 96% of *Alt-a-1* regions of *Alternaria,* resulting in 547 bp sequences. Sequence alignment analysis revealed 99.8% of the *Alternaria dauci* of length for *Alt-a-1* gene sequences. 

**Figure 5:**
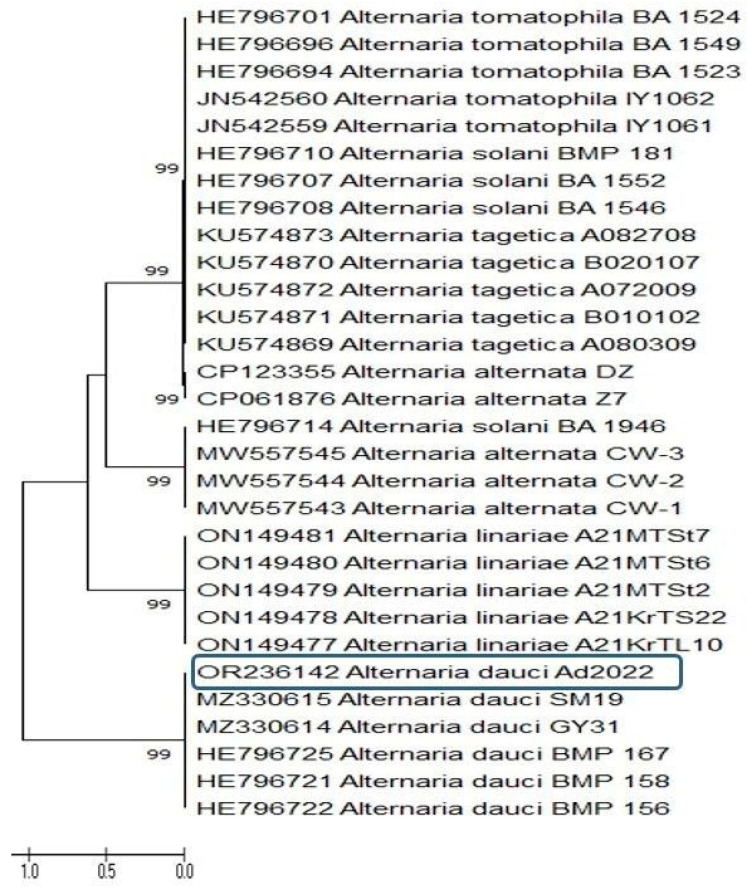
A phylogenetic tree, using sequence analysis of a concatenated sequence from the *Alt-a-1* gene, shows the *Alternaria dauci* (GenBank number; OR236142, black box) among the similar sequences *Alternaria* spp. found in the GenBank database. Bootstrap of 1000 replications; external group.

Accordingly, the results of the phylogenic tree sequence analysis of *A. dauci* Ad2022 with the *Alternaria* spp. have a fan shape (Figure 5), showing two main clusters; the first main cluster contains isolate Ad2022 and five accession numbers of other *A. dauci,* while the second main cluster contains the other *Alternaria* spp. (e.g., *A. tagetica*, *A. tomatophila, A. linariae, A. solani*, and *A. alternata*). We conclude that the strain (Ad2022) has the highest similarity to *A. dauci*. The *Alt-a-1* gene sequence confirms the previous morphological and ITS identifications. 

Phylogenetic investigation of the DNA sequence of the *Alternaria major* allergen (*Alt-a-1*) gene region has been utilized for the characterization of the fungus [66]. Because the cryptic diverse species were described, the gene alone was not sufficient to identify *Alternaria* spp. [67]. Additionally, the ITS approach, together with gene identification, was recommended to demonstrate accurate identification of *Alternaria* [68]. Nine gene regions were used to revise the *Alternaria* spp. [69]. The *Alt-a-1* gene was found to be dominant in the majority of Alternaria spp.; thus, identifying the Alternaria species based on such genes, in addition to morphological features as well as ITS regions, is difficult but useful for accurate identification [30].

Curiously, our well-identified sequences of ITS and the *Alt-a-1* gene show a deep correlation with the other similar *Alternaria* spp. strains in the GenBank. Further, the molecular identification was similar to the morphoslogical one, confirming the classification position of the pathogenic fungus as Eukaryota; Kingdom: Fungi; Division: Ascomycota; Class: Dothideomycetes; Order: Pleosporales; Family: Pleosporaceae; Genus: *Alternaria*; Species: *A. dauci*.

## 4. Conclusions

In summary, this comprehensive investigation represents the first in-depth examination of *A. dauci* on coriander plants. Our study on the newly detected coriander leaf spot highlights the imminent threat of this disease on a global scale. Morphological identification confirms the causal agent as *A. dauci*. Our analysis of the fungal enzymatic profile reveals extensive lytic activity, which could serve as a potential avenue for infection. This lytic feature may also be indicative of the activation of virulence factors in certain fungal species. Furthermore, the current study primarily studies genetic variation using two genes (ITS ribosomal RNA and *Alt-a-1* gene analysis), and the molecular identification aligns with the morphological findings. The unprecedented lytic and molecular characteristics of this pathogen could pave the way for outbreaks in various regions.

Of particular concern is the pathogen’s appearance in Egypt, marking its first occurrence in the African and Middle Eastern regions, which raises the possibility of it spreading to other areas. We must remain vigilant and proactive in monitoring and managing this threat. Nevertheless, we encourage further research to explore the potential link between climatic changes and the emergence of this pathogen in new regions.

## Figures and Tables

**Table 1:** *Alt-a-1* gene sequences of related isolates with similarity percentages of more than 97.5%, downloaded from the GenBank database.

Isolate	Accession Number	E-Value	Query Coverage, %	Similarity, %
*Alternaria dauci*	MZ330615.1	0.0	96	99.8
*Alternaria dauci*	MZ330614.1	0.0	96	99.8
*Alternaria dauci*	HE796725.1	0.0	96	99.8
*Alternaria dauci*	HE796721.1	0.0	96	99.8
*Alternaria dauci*	HE796722.1	0.0	96	99.8
*Alternaria dauci*	HE796723.1	0.0	96	99.8
*Alternaria dauci*	KJ732976.1	0.0	94	99.4
*Alternaria tagetica*	KU574873.1	0.0	94	98.8
*Alternaria tagetica*	KU574872.1	0.0	94	97.5
*Alternaria tagetica*	KU574871.1	0.0	94	97.5

## Data Availability

All relevant data are within the paper.
